# The Importance of Ethics in Modern Universities of Technology

**DOI:** 10.1007/s11948-019-00164-6

**Published:** 2019-12-24

**Authors:** Behnam Taebi, Jeroen van den Hoven, Stephanie J. Bird

**Affiliations:** 1 Department of Values, Technology and Innovation, Faculty of Technology, Policy and Management, Delft University of Technology, PO 5015, 2600 GA Delft, The Netherlands; 2 Massachusetts Institute of Technology, PO Box 2007, Wrentham, MA USA

The twenty-first century will pose substantial and unprecedented challenges to modern societies. The world population is growing while societies are pursuing higher levels of global well-being. The rise of artificial intelligence (AI) and autonomous systems, increasing energy demands and related problems of climate change are only a few of the many major issues humanity is facing in this century. Universities of technology have an essential role to play in meeting these concerns by generating scientific knowledge, achieving technological breakthroughs, and educating scientists and engineers to think and work for the public good.

The aim of this Special Issue of *Science and Engineering Ethics* is to examine some of the ethical issues that arise for institutions of higher education in the field of engineering and applied science in meeting these challenges. In so doing, it highlights two specific areas. First, it considers the ethical issues that arise for institutions of higher education in the area of engineering and applied science. Its focus is on specific issues at universities of technology, more specifically on the relationships both of individual academic researchers, and of institutions themselves, with industrial partners, commerce and innovation in for-profit organizations. The second area of focus is the matter of educating a new generation of engineers and scientists so that they will be equipped to deal with the future challenges that mankind faces, while also observing the highest moral standards of academic conduct and research integrity.

In the arena of AI, events in 2019 have brought to light a number of cases that highlight increased sensitivity within and beyond the academic community to morally problematic crossover and interconnections between the corporate world and academia. For example, the director of the Massachusetts Institute of Technology (MIT) Media Lab stepped down as a result of significant controversy regarding the acceptance of funding from Jeffrey Epstein, the disgraced financier who faced sex trafficking charges (Tracy and Hsu 2019). In addition, Google’s AI ethics council (the Advanced Technology External Advisory Council), with members from academia, the corporate world, and think tanks, was dissolved immediately after the membership of an anti-LGBT advocate on the committee led to controversy among Google workers and in the media (Levin 2019). Moreover, because the stakes and commercial interests in AI research have become so significant, there are a variety of efforts underway to influence both the course of research into the ethics of AI, and the future of regulation and governance. Facebook has made a $7.5 million (US) donation to the Technical University of Munich, establishing a new center devoted to the ethics of artificial intelligence (Kahn 2019). The private equity firm, Blackstone, has donated $180 million US dollars to Oxford University for both an ethics center, and for humanities and social sciences research into AI (Williams 2019; Reuters 2019). Further, the Wallenberg foundation is funding ethics and AI research in Sweden (WASP-HS 2019).

This Special Issue has grown out of a workshop on ‘Science and Integrity in the Modern University’ that was held at Delft University of Technology in the Netherlands in 2013.^1^ This workshop was organized in order to reflect on the so-called ‘Valorization task’ (i.e., the assignment to contribute effectively in addressing the societal challenges posed by technology) that universities, and more specifically universities of technology, are increasingly expected to assume as one of their core tasks.^2^ The term ‘valorization’ refers to a process of facilitating knowledge transfer and, ideally, it creates benefits for society because scientific knowledge can then be translated into tangible results. While valorization is not necessarily a new phenomenon for universities of technology, the close and extensive collaboration between independent researchers working in academia and industry can raise intricate ethical questions. These questions need to be fully acknowledged and addressed. An *ethics infrastructure* is therefore indispensable when it comes to dealing with ethical issues that are specific for universities of technology. These issues are not only relevant to research, but they should also have a significant influence in shaping the education of future engineers. This Special Issue is particularly aimed at considering both concerns for universities of technology such as the issues and potential conflicts associated with research funding, and also challenges associated with teaching broadly-oriented future scientists and engineers.

This issue is organized such that each original research article is accompanied by a commentary. Comment pieces are not critiques of the arguments in the research article, nor are they summaries of the primary article. Rather, they are short essays on the same or a related topic aimed at providing the reader with an additional perspective. Further, this Special Issue has a very broad international viewpoint. Authors report on and reflect fascinating developments in the US, the Netherlands and Spain, but also in China and South Africa.

## Ethical Challenges in Research at Technical Universities

Scientific research is expected to be in the service of society and certainly not to be at odds with the responsible pursuit of intelligent solutions to its problems. Among research funding agencies, it is commonly assumed that an important indicator for measuring social relevance is the willingness of industry to invest in research. In many countries, there is an upsurge in government policies aimed at encouraging academic-industry collaborations. Universities are expected to facilitate knowledge transfer to industry by means of systematic collaborations. Indeed, in some instances governmental funding schemes for independent research depend on co-funding by industry. The rationale behind these policies is that research in which industry is willing to invest is marketable and, hence, socially relevant. Especially for universities of technology, a university’s research income increasingly depends on these collaborations. This raises the question of how to design and implement institutional arrangements in order to anticipate and deal with potential conflicts of interest that might occur, and to address the effects these could have on the independence of scientific judgment.

The emphasis on interactions between the academic domain and the market domain raises concerns that have been discussed by philosopher Michael Walzer, author of *Spheres of Justice* (Walzer 1983). He has argued that different spheres in society have their own normative logics. Many societies try to prevent the intermingling of the normative logics (e.g., expressed in the governance of institutions) of these separate spheres, that is, they attempt to ensure that criteria for the distribution of goods in one sphere are not used to allocate advantages in another sphere. In order to prevent this cross contamination, Western, democratic societies typically practice what Walzer calls ‘the art of separation’ of spheres. For example, in Western, liberal democracies constraints are put on what money can buy and it is a widely held view that appointments to political office should be kept separate from commercial considerations; that is, money should not be allowed to buy influence or power in the political sphere. There are norms and rules that govern the allocation of political responsibility, namely democratic elections. Further, eligibility for medical treatment should be kept separate from someone’s status in the political sphere and therefore, priority on a waiting list for heart surgery should depend on ‘need’, not on wealth or status. Walzer describes how some societies have blocked exchanges at the boundaries of social spheres so that family relationships cannot facilitate admission to a university, or that a university degree cannot influence eligibility to receive health care. Similarly, boundaries between the sphere of science and the market should be critically monitored so that financial gain does not compromise independent scientific judgment. Norms concerning proper conduct in science cannot be replaced by norms governing market behavior and the profit motive. The pursuit of scientific truth, or of a better understanding of the world, should in principle be kept separate from commercial benefits associated with it.

Modern scientists run the risk of becoming the victim of role confusion and conflicts of interest similar to the company doctor who inhabits two worlds i.e., the doctor’s clinical judgment runs the risk of being compromised or undermined by his allegiance to economic thinking within the company. The scientist in the age of commerce could similarly acquire some of the tragic features of the company doctor—normative confusion by design—unless the scientist’s responsibilities and loyalties are clearly defined and separated and the interplay is made transparent. A university’s ethics infrastructure should promote and facilitate this separation and avoid any semblance of conflicts of interest. Such infrastructure should at least include arrangements, rules and institutions to facilitate raising an ethical dilemma (e.g., a breach of integrity or a question about the use of human subjects in research) and to enable addressing this issue further.

In ‘Values in University-Industry Collaborations: The Case of Academics Working at Universities of Technology’, Rafaela Hillerbrand and Claudia Werker discuss the challenges of scholars working at universities and in industry (Hillerbrand and Werker 2019). They specifically focus on the role of an individual scholar, who may run into serious conflicts when working on joint university-industry projects. While universities aim to disseminate knowledge, industry aims to appropriate knowledge. This *role confusion* can lead to ethically problematic and complex situations and conflicts of interests. In his commentary, ‘The Need for a Code of Conduct for Research Funders’, Bert van Wee argues that the attention only on researchers in the ethics literature is insufficient: the focus needs to expand to include a code of conduct for funders of research (van Wee 2019). Van Wee’s commentary provides tangible recommendations such as ‘policy relevant research should not be contracted and supervised by a client with an interest in the outcomes’, and ‘policy relevant research should always be examined by an independent institute’.

In his contribution ‘Institutional Conflicts of Interest in Academic Research’, David Resnik extends discussions of conflicts of interest to the level of institutions (Resnik 2015). For example, institutional officials may have individual financial relationships that may inappropriately influence decision-making, and, together or separately, can give rise to an institutional conflict of interest (iCOI). In their commentary, ‘Current Perspectives Regarding Institutional Conflict of Interest’, Ann Nichols-Casebolt and Francis Macrina argue that academic institutions must develop strategies to remediate the unique challenges in iCOI, including clarifying the definition of iCOI and implementing a well-designed electronic database for reporting and managing iCOI across multiple leadership constituencies (Nichols-Casebolt and Macrina 2015).

Seumas Miller, in his contribution ‘Whither the University?: Universities of Technology and the Problem of institutional Purpose’, addresses the need to provide an appropriate normative conception of the modern university (Miller 2019). Such conception could help to admit differences between universities of technology and other universities. Building on the teleological normative theory of social institutions which implies that universities are to be considered organizations that provide collective goods by means of joint activity, Miller discusses the fundamental collective good(s) that universities of technology ought to provide. He argues that the absence of a normative conception is partially masked by the process of institutional evolution that has actually been taking place at universities. In her commentary, ‘The Survival Imperative’, Stephanie Bird delves further into the evolutionary requirement that a species pass on its ‘survival knowledge’ to the next generation (Bird 2019). Humans as a species tend to be too clever, powerful, ignorant and arrogant for our own good, and the good of the planet. It is essential that humanity and its societies determine how to more effectively teach future generations the key information they need to address their limitations and survive.

The last two pieces in this part focus on fascinating new efforts in China and South Africa. In their paper, ‘Ethics “Upfront”: Generating an Organizational Framework for a New University of Technology’, Penelope Engel-Hills, Christine Winberg and Arie Rip highlight an expectation in post-apartheid higher education in South Africa that technikons (institutions similar to the British polytechnics) will be/should be converted to universities of technology (Engel-Hills et al. 2019). They discuss one of the new South African universities of technology as a case study and, more specifically, the opportunity to build a new university such that ethics could be placed ‘upfront’, rather than coming as an afterthought. This ethics upfront approach requires constructing an organizational framework that makes ethical issues integral to management and decision-making processes. In their commentary ‘Development of Ethics Education in Science and Technology in Technical Universities in China’, Qian Wang and Ping Yan introduce the specific situation and characteristics of ethics education in science and technology at Chinese technical universities (Wang and Yan 2019). China’s ethics education in science and technology in China’s five technical universities (also known as the 5TU) emphasizes the use of traditional ideological and cultural resources and practical cases. Teaching methods combine traditional Chinese ethics with non-Chinese experience and teaching methods, and aim at cultivating students’ ability to solve ethical problems in the real world.

## Challenges in Teaching Ethics to Engineers and Scientists

The second part of the special issue contains papers that mainly focus on teaching endeavors. In their piece ‘Ethics Across the Curriculum: Prospects for Broader (and Deeper) Teaching and Learning in Research’, Carl Mitcham and Elaine Englehardt assert that the movements to teach the responsible conduct of research (RCR) and engineering ethics at technological universities is not receiving enough scholarly attention; they argue that RCR should be seen as a part of the broader ethics across the curriculum (EAC) movement that is receiving more scholarly attention (Mitcham and Englehardt 2016). The authors compare EAC initiatives at different universities, including the successful one at Utah Valley University that gave birth to EAC as a scholarly movement, and the one at the Colorado School of Mines that manifests continuing institutional resistance to EAC. In their commentary, ‘Teaching Engineering Ethics to PhD Students: A Berkeley–Delft Initiative’, Behnam Taebi and William Kastenberg draw a similar comparison between the University of California at Berkeley and Delft University of Technology (Taebi and Kastenberg 2016). The commentary highlights a variety of academic and institutional challenges at these two universities, when jointly teaching a graduate engineering ethics course first at UC Berkeley and later at Delft University. The authors argue that both a bottom-up approach at the level of the faculty and as a joint research and teaching effort, and a top-down approach that includes recognition by a University’s administration and the top level of education management, are needed for successful and sustainable efforts to teach engineering ethics.

Mary Sunderland in her ‘Using Student Engagement to Relocate Ethics to the Core of the Engineering Curriculum’ considers the core problem of *perception* with engineering ethics education: while ethics is meant to be a central component of today’s engineering curriculum, it is often perceived as a marginal requirement to be fulfilled (Sunderland 2013). There is further a mismatch between the faculty’s perceptions of ethics as emphasizing the nuances and complexity of engineering ethics, while students tend to perceive ethics as laws, rules, and codes that must be memorized and reproduced. Sunderland describes a student engagement approach to pedagogy that includes students as active participants in curriculum design, which could help relocate ethics from the periphery to the core of the engineering curriculum. In his commentary ‘Ethics and the UN Sustainable Development Goals: The Case for Comprehensive Engineering’, Jeroen van den Hoven explores another important aspect in engineering curricula that should accommodate ‘comprehensive engineering’, as an approach that could help to accommodate ethical coherence, consilience of scientific disciplines, and cooperation between parties (van den Hoven 2016). Comprehensive engineering is key if engineers are to adequately and responsibly respond to the global problems that the world is facing, such as those formulated in the United Nations Sustainable Development Goals.

Alejandra Boni, José Javier Sastre and Carola Calabuig, in their article ‘Educating Engineers for the Public Good Through International Internships: Evidence from a Case Study at Universitat Politècnica de València’, discuss a different approach to creating awareness among engineering students about their social responsibility through an internship program that places engineering students in countries of Latin America in order to expose them to the implications of being a professional in society in a different cultural and social context (Boni et al. 2015). An integral part of this program is a reflection on the dynamic relationship between technology and society by creating space before and during the internship, and upon the return of the students, to discuss and collectively reflect upon their lived experience. Colleen Murphy and Paolo Gardoni, in their commentary ‘Understanding Engineers’ Responsibilities: A Prerequisite to Designing Engineering Education’, emphasize that all activities in engineering education, including study time abroad as well as internships, must be based on a comprehensive understanding of engineers’ responsibilities (Murphy and Gardoni 2017). Globalization has implications for these responsibilities and international internships can play an important role in fostering the requisite moral imagination of engineering.

## Footnotes

1 The editors of this issue were involved in the workshop at Delft University as organizers (Jeroen van den Hoven and Behnam Taebi) and as a speaker (Stephanie Bird). In addition, two of the authors of the articles published here (i.e., David Resnik and Ann Nichols-Casebolt) were also speakers at the workshop.

2 This is a popularized term in the academic setting in several parts of the world, including the Netherlands. More on the meaning of this term can be found on the website of the Association of Dutch Universities: https://www.vsnu.nl/en_GB/f_c_valorisatie.html (Retrieved on 26 November 2019).
